# Vitamin D Receptor Gene Polymorphisms Are Associated With Leprosy in Southern Brazil

**DOI:** 10.3389/fimmu.2019.02157

**Published:** 2019-10-04

**Authors:** Afonso Carrasco Pepineli, Hugo Vicentin Alves, Bruna Tiaki Tiyo, Luciana Conci Macedo, Lorena Visentainer, Quirino Alves de Lima Neto, Joana Maira Valentini Zacarias, Ana Maria Sell, Jeane Eliete Laguila Visentainer

**Affiliations:** ^1^Laboratory of Immunogenetics, Department of Basic Health Sciences, Maringá State University (UEM), Maringá, Brazil; ^2^Department of Medicine, Faculty of Medical Sciences, Campinas State University (UNICAMP), São Paulo, Brazil

**Keywords:** multibacillary, paucibacillary, genetic polymorphism, case-control studies, gene frequencies, biological markers

## Abstract

Vitamin D, together with its nuclear receptor (VDR), plays an important role in modulating the immune response, decreasing the inflammatory process. Some polymorphisms of the *VDR* gene, such as *Bsm*I (G>A rs1544410)*, Apa*I (G>T rs7975232), and *Taq*I (T>C rs731236) could affect its stability and mRNA transcription activity, while *Fok*I T>C (rs2228570) gives a truncated protein with three fewer amino acids and more efficiency in binding vitamin D. This study evaluated these four polymorphisms in the immunopathogenesis of leprosy in 404 patients and 432 control individuals without chronic or infectious disease in southern Brazil. When analyzing differences in the allele and genotype frequency of polymorphisms between patients (leprosy *per se*, multibacillary, and paucibacillary clinical forms) and controls, we found no statistically significant association. Regarding haplotype analysis, the bAt haplotype was associated with protection from leprosy *per se* (*P* = 0.004, OR = 0.34, CI = 0.16–0.71) and from the multibacillary clinical form (*P* = 0.005, OR = 0.30, CI = 0.13–0.70). In individuals aged 40 or more years, this haplotype has also showed protection against leprosy *per se* and multibacillary (OR = 0.26, CI = 0.09–0.76; OR = 0.26, CI = 0.07–0.78, respectively), while the BAt haplotype was a risk factor for leprosy *per se* in the same age group (OR = 1.34, CI = 1.04–1.73). In conclusion, despite having found no associations between the *VDR* gene polymorphisms with the development of leprosy, the haplotypes formed by the *Bsm*I, *Apa*I, and *Taq*I polymorphisms were associated with leprosy *per se* and the multibacillary clinical form.

## Introduction

Leprosy is a chronic granulomatous disease caused by *Mycobacterium leprae*, which mainly affects macrophages of the skin, Schwann cells of the peripheral nerve, and eventually other organs and systems (1). Leprosy is among the three neglected diseases with the highest prevalence worldwide. According to the World Health Organization (WHO), in 2017 its incidence reached 210,671 new cases of the disease worldwide. In Brazil, the number of new cases registered in that year was more than 26,000. This sets Brazil at the second place in the ranking of countries with the largest number of leprosy cases in the world (2).

There is considerable clinical variability among leprosy patients once *M. leprae* infection evokes distinct T cell responses in humans. The classification of leprosy according to immunity includes the following clinical forms: tuberculoid (TT), lepromatous (LL), borderline tuberculoid (BT), borderline borderline (BB), and borderline lepromatous (BL) (3, 4). However, based on the number of skin lesions, the patients can be classified as paucibacillary (PB) and multibacillary (MB) leprosy cases, according to WHO (5, 6). The type of immune response may determine the clinical form, as well as the resistance or susceptibility to the disease. The TT form (also classified as paucibacillary) is characterized by a small number of hypopigmented, well-bordered, anesthetic skin lesions with a low bacillary load, early peripheral nerve impairment, and a T-helper 1 (Th1)-mediated immune response. On the other hand, in form LL (therefore referred to as multibacillary), there is a prevalence of the Th2-mediated immune response, which leads to numerous infiltrated skin lesions displaying high bacillary loads, impaired peripheral nerves, and possible involvement of internal organs (7, 8). However, the predominance of one type of immune response does not mean that cytokines from the other response profiles are not being produced (9).

Many factors can modulate the type of immune response developed by the leprosy patient, such as vaccination with BCG, nutritional status, degree of exposure to *M. leprae*, and infections by other microorganisms. Besides, leprosy susceptibility can also be influenced by genetic factors (10). As vitamin D metabolizing enzymes and vitamin D receptors are present in many cell types, including various immune cells such as antigen-presenting cells, T cells, B cells, and monocytes, these molecules are important targets in the study of polymorphisms that can modulate the immune response against pathogens (11). Among the immunomodulatory roles of vitamin D is the inhibition of MHC class II, CD40, CD80, and CD86, which leads to the blocking of the Th1 response and activation of regulatory T cells (12). Nuclear vitamin D receptor (VDR) is an intracellular polypeptide that binds to the active vitamin D metabolite, 1,25-Dihydroxyvitamin D_3_ (1,25-(OH)_2_-D_3_), and then interacts with the chromatin, producing a variety of genomic effects (13, 14), such as pleiotropic regulation of human physiology, protection of the cardiac system, cancer prevention, and modulation of the immune system (15, 16). Vitamin D is a direct and indirect regulator of the immune system. The VDR are expressed in T and B cells, dendritic cells, and cells of the monocyte/macrophage lineage (17, 18). Vitamin D acts in suppressing the development of several autoimmune diseases and tissue damage (19–22). The effects of vitamin D on murines and humans include: the development of dendritic cells and T regulatory cells; inhibition of T cell proliferation; inhibition of IFN-γ and IL-17 production; and the induction of IL-4 expression (23, 24). Furthermore, the VDR activation leads to the inhibition of both maturation and proliferation of activated B cells and limits antibody production (25).

The *VDR* locus is at chromosome 12q13.1 and spans over 75 kb of genomic DNA. The human gene has three transcript variants which encode the Vitamin D3 receptor isoforms VDRA and VDRB1. The first exons of the *VDR* gene make up the leader sequence and the exons 2–9 encode the structural portion of the gene product (26–29). There are some polymorphisms located near the 3'UTR region of the *VDR* gene that may affect mRNA stability and translation. These polymorphisms are: *Bsm*I G>A (rs1544410, G allele designated “b” and A allele designated “B”); *Apa*I G>T (rs7975232, G allele designated “a” and T allele designated “A”), and *Taq*I T>C (rs731236, T allele designated “T” and C allele designated “t”) (30). Another polymorphism known as *Fok*I T>C (rs2228570, T allele designated “f” and C allele designated “F”), located within the start codon in exon 2 of *VDR*, gives a truncated protein with three fewer amino acids. The F allele gives rise to the variant protein, which is more efficient in mediating vitamin D action (31, 32). Therefore, considering the role that the active metabolite of VDR exert in the mechanisms of immunity, this study evaluated the association of SNPs (single nucleotide polymorphisms) of the *VDR* gene (*Fok*I, *Bsm*I, *Apa*l, and *Taq*I) with the immunopathogenesis and clinical forms of leprosy.

## Materials and Methods

### Study Population

A total of 404 patients (230 men and 174 women) with leprosy from the northwestern region of Paraná, southern Brazil (22° 29′ 30″-26° 42′ 59″ S and 48° 02′ 24″-54° 37′ 38″ W), aged 10–93 years (55.00 ± 13.95), and diagnosed by clinical examination, bacilloscopy, and biopsy were evaluated. Written informed consent was obtained from the participants in this study, including the parents of participants under the age of 16.

In accordance with previous studies, investigations should not be restricted to a sub-analysis of overall leprosy, but should instead contrast multibacillary (MB) and paucibacillary (PB) individuals (6). Thus, patients were reclassified to MB (*n* = 310) and PB (*n* = 86). The control group consisted of 432 non-consanguineous individuals from the same region as the patients, and they declared that they did not present any chronic or infectious diseases. Of these, 231 were female and 201 were male, and the age of the controls ranged from 16 to 105 years (50.69 ± 18.07). The characteristics of patients and controls are described in Table 1. All participants were classified as a mixed population from southern Brazil, according to the distribution already described in populations of Paraná (33): predominantly of European origin (80.6%), with a smaller contribution of African (12.5%) and Amerindian (7.0%).

**Table 1:** Profile of leprosy patients and controls.

		**Leprosy *per se***	**PB**	**MB**	**Controls**
		***N* = 404**	***N* = 86**	***N* = 310**	***N* = 432**
**Variables**		***n* (%)**	***n* (%)**	***n* (%)**	***n* (%)**
Age	≥40	343 (84.90)	74 (85.05)	263 (84.84)	373 (86.34)
	<40	61 (15.10)	12 (13.95)	47 (15.16)	59 (13.66)
Gender*	Male	230 (56.93)	34 (39.53)	190 (61.29)	201 (46.53)
	Female	174 (43.07)	52 (60.47)	120 (38.71)	231 (53.47)

* *Statistically significant difference between leprosy per se patients and controls (P = 0.003, OR = 1.52, 95% CI = 1.15–2.02); PB, Paucibacillary; MB, Multibacillary; N, population size; n, number of individuals; %, percentage*.

### Genotyping

The genotyping of the samples with respect to the *VDR* gene polymorphisms: *Fok*I T>C (rs2228570), *Bsm*I G>A (rs1544410), *Apa*I G>T (rs7975232), and *Taq*I T>C (rs731236) was performed by PCR-RFLP (polymerase chain reaction) (34, 35) with modifications. The technique was validated by direct sequencing of 15 samples for each variant for the SNPs, using BigDye™ Terminator v3.1 Cycle Sequencing Kit (ThermoFisher). Restriction enzymes were used according to the manufacturer's recommendations: *Fok*I # R0109S (BioLabs®Inc), *Taq*I # ER0671 (ThermoFisher), *Apa*I # ER1411 (ThermoFisher), and *Mva*1269I # ER0961 (*Bsm*I) (ThermoFisher). After restriction enzyme digestion, the amplification products were subjected to 2% agarose gel electrophoresis.

### Statistical Analysis

The sample size was calculated using the QUANTO software (www.biostats.usc.edu/software), aiming to reach a power of 80%. The SNPStats software (https://www.snpstats.net/start.htm) and OpenEpi program Version 3.01 (https://www.openepi.com/Menu/OE_Menu.htm) were used to determine the allelic, genotypic, and haplotypic frequencies of the *VDR* gene polymorphisms, and to verify the statistical differences between the groups. The association tests were performed for codominant, dominant, recessive, over dominant, and log-additive genetic inheritance models (36). Haplotype frequency estimates were carried out using expectation–maximization algorithms. Odds ratios with 95% confidence intervals were deemed necessary only for significant *P*-values. All tests were carried out using a significance level of 5%. Genotype frequency distributions were evaluated to ensure Hardy-Weinberg equilibrium for all genes in the populations.

## Results

In this case-control study, allele and genotype frequency distributions of *Fok*I T>C (rs2228570), *Bsm*I G>A (rs1544410), *Apa*I G>T (rs7975232), and *Taq*I T>C (rs731236) SNPs were analyzed in a total of 404 patients with leprosy *per se* (of these, 310 were classified as multibacillary, 86 as paucibacillary, and 8 individuals were not classified) and 432 controls. The distribution of the genotype frequencies for all analyzed genes was consistent with the Hardy-Weinberg equilibrium (*P* > 0.05). To avoid bias, the gender was used as an adjustment covariate between leprosy *per se* and the control because of the non-pairing between the groups. Differences in the allele and genotype frequency distributions were not observed between patients (leprosy *per se*, MB, and PB clinical forms) and controls in linear analyses in the recessive, dominant or codominant inheritance models. There was also no statistically significant difference when comparing the PB and MB clinical forms. The *Fok*I, *Bsm*I, *Apa*I, and *Taq*I genotype and allele frequency distributions are summarized in Table 2.

**Table 2:** Genotype and allele frequency distributions for *Fok*I, *Bsm*I, *Apa*I, and *Taq*I polymorphisms in leprosy *per se*, PB, and MB clinical forms patients and controls.

	**Leprosy *per se***	**PB**	**MB**	**Controls**
	***N* = 404**	***N* = 86**	***N* = 310**	***N* = 432**
**Genotype and alleles**	***n* (%)**	***n* (%)**	***n* (%)**	***n* (%)**
***Fok*****I (rs2228570)**				
F/F	176 (43.6)	42 (48.8)	127 (41)	191 (44.2)
F/f	168 (41.6)	32 (37.2)	135 (43.5)	195 (45.1)
f/f	60 (14.8)	12 (13.9)	48 (15.5)	46 (10.7)
F	520 (64.4)	116 (67.0)	389 (63.0)	577 (66.8)
f	288 (35.6)	56 (33.0)	231 (37.0)	287 (33.2)
***Bsm*****I (rs1544410)**				
B/B	57 (14.1)	12 (13.9)	44 (14.2)	52 (12.0)
B/b	199 (49.3)	44 (51.2)	153 (49.4)	202 (46.8)
b/b	148 (36.6)	30 (34.9)	113 (36.5)	178 (41.2)
B	313 (38.7)	68 (40.0)	241 (39.0)	306 (35.4)
b	495 (61.3)	104 (60.0)	379 (61.0)	558 (64.6)
***Apa*****I (rs7975232)**				
A/A	129 (31.9)	30 (34.9)	97 (31.3)	131 (30.3)
A/a	199 (49.3)	43 (50)	151 (48.7)	214 (49.5)
a/a	76 (18.8)	13 (15.1)	62 (20.0)	87 (20.1)
A	457 (56.6)	103 (60.0)	345 (56.0)	476 (55.1)
a	351 (43.4)	69 (40.0)	275 (44.0)	388 (44.9)
***Taq*****I (rs731236)**				
T/T	153 (37.9)	32 (37.2)	115 (37.1)	184 (42.6)
T/t	203 (50.2)	44 (51.2)	158 (51)	198 (45.8)
t/t	48 (11.9)	10 (11.6)	37 (11.9)	50 (11.6)
T	509 (63.0)	108 (63.0)	388 (63.0)	566 (65.5)
t	299 (37.0)	64 (37.0)	232 (37.0)	298 (34.5)

*PB, Paucibacillary; MB, Multibacillary; N, population size; n, number of individuals; %, percentage*.

The *Bsm*I, *Apa*I, and *Taq*I polymorphisms were in linkage disequilibrium (D = 0.93, 0.83, and 0.92, respectively). When we evaluated the influence of the haplotypes formed by the *Bsm*I, *Apa*I, and *Taq*I polymorphisms of the *VDR* gene, the haplotype bAt was associated with protection against leprosy *per se* and the MB clinical form (*P* = 0.004, OR = 0.34, CI = 0.16–0.71; *P* = 0.005, OR = 0.30, CI = 0.13–0.70, respectively), as shown in Table 3. When the haplotype was associated with age, the bAt haplotype showed protection against leprosy *per se* and MB in individuals aged 40 or more years (OR = 0.26, CI = 0.09–0.76; OR = 0.24, CI = 0.07–0.78, respectively). Whereas, the BAt haplotype was a risk factor for leprosy *per se* in the same age group (OR = 1.34, CI = 1.04–1.73). The haplotype and age cross-classification interaction are shown in Table 4.

**Table 3:** Haplotypes formed by the *Bsm*I, *Apa*I, and *Taq*I polymorphisms of the *VDR* gene evaluated in all individuals (leprosy *per se* patients and controls) and MB clinical form (MB patients and controls).

***Bsm*I, *Apa*I, and *Taq*I**	**All individuals frequencies**	***P***	**OR (95% CI)**	**MB and controls frequencies**	***P***	**OR (95% CI)**
**Haplotypes**	***N* = 836**			***N* = 742**		
baT	0.4207	Ref.		0.4229	Ref.	
BAt	0.3167	0.05		0.3137	0.09	
bAT	0.1719	0.6		0.1686	0.38	
BAT	0.0416	0.06		0.0414	0.036	0.53 (0.29–0.96)*
bAt	0.0279	0.004	0.34 (0.16–0.71)	0.0294	0.005	0.30 (0.13–0.70)
Rare	0.0213	0.001	0.13 (0.04–0.45)	0.0238		

MB, Multibacillary; N, population size; P, P value; OR, odds ratio; CI, confidence interval.

* *This result was disregarded because the CI is so near to one*.

**Table 4:** Haplotypes formed by the *Bsm*I, *Apa*I, and *Taq*I polymorphisms of the *VDR* gene evaluated in all individuals (leprosy *per se* patients and controls) and MB clinical form (MB patients and controls) and age cross-classification interaction.

	**All individuals frequencies**			**MB and controls frequencies**		
***Bsm*I, *Apa*I, and *Taq*I**		**Age < 40**	**Age ≥ 40**		**Age < 40**	**Age ≥ 40**
**Haplotypes**	**(*N* = 836)**	**OR (95% CI)**	**OR (95% CI)**	**(*N* = 742)**	**OR (95% CI)**	**OR (95% CI)**
baT	0.4207	Ref.		0.4229	Ref.	
BAt	0.3167			0.3138		1.34 (1.04–1.73)
bAT	0.1719			0.1686		
TAB	0.0416			0.0414		
bAt	0.0279		0.26 (0.09–0.76)	0.0294		0.24 (0.07–0.78)
rare	0.0213			0.0238		

*MB, Multibacillary; N, population size; P, P value; OR, odds ratio; CI, confidence interval*.

## Discussion

This case-control study investigated the genotypic and allelic frequencies of certain *VDR* gene polymorphisms in leprosy patients and controls without the disease, in order to evaluate whether these polymorphisms could act as factors of susceptibility or resistance to the disease or to a specific clinical form. When analyzing differences in the allele and genotype frequency distributions between patients (leprosy *per se*, MB, and PB clinical forms) and controls, we found no statistically significant association.

Consistent with our results, other studies have also found no association between the *Apa*I polymorphism and leprosy (37). The *Bsm*I polymorphism has also showed no statistically significant association with leprosy in a couple of studies (37, 38). A recent meta-analysis of this polymorphism and tuberculosis showed that the b-allele was a risk factor for disease development, but this was only observed in the Asian population (39). Regarding *Bsm*I, *Apa*I, and *Taq*I polymorphisms, it is not clear whether they have an individual effect on the expression or function of the VDR. It is possible that the associations found for these polymorphisms in the various diseases studied, if they actually exist, occurred due to a linkage disequilibrium with a polymorphism that has a functional effect on these diseases (37). A recent systematic review of VDR and leprosy suggests that such a large diversity of results would be a consequence of ethnic heterogeneity, sample size used, design of each study, and also influences from other regions of the gene that have not been studied yet, as well as the likelihood of bacillus virulence being distinct in the different geographic regions where the studies took place (40).

The results concerning haplotypes are in agreement with the hypothesis that the *Bsm*I, *Apa*I, and *Taq*I polymorphisms are not directly related to leprosy. However, it is a significant result because it shows that these SNPs may be in linkage disequilibrium with another functional polymorphism. We have analyzed five haplotype alleles in our study, of which haplotypes 1 (baT; 42%), 2 (BAt; 32%), and 3 (bAT; 17%) were the most frequent and were similar to the ones identified by Uitterlinden et al. (41), haplotype 1 (baT; 48%), 2 (BAt; 39%), and 3 (bAT; 11%). Despite our finding of an association of the haplotype bAt with protection against leprosy *per se* and the MB clinical form, there is no evidence in the literature about the influence of this haplotype in any disease. Moreover, this association could be due to the low frequency of this haplotype in our individuals.

In contrast, the haplotype 2 (BAt) was associated with risk to leprosy *per se* when the patients were divided by age. Although studies do not agree on which VDR haplotypes are related to susceptibility or protection for various diseases, the fact that BAt shows susceptibility to leprosy can be explained due to a strong linkage disequilibrium between the *Bsm*I, *Apa*I, and *Taq*I haplotypes and the poly(A) variable number of tandem repeats (VNTRs) in the 3′UTR of the *VDR* gene. The poly(A) VNTR polymorphism can be characterized as bi-allelic, and subjects can be classified as having alleles with short or long poly(A) stretches. There is a strong linkage between the haplotype 1 (baT) and the long poly(A) stretch (*n* = 18–24, long or L alleles), while the haplotype 2 (BAt) is in linkage to the short poly(A) stretch (*n* = 13–17, short or S alleles). There seems to be a trend for the BAt haplotype to display overall somewhat higher levels of mRNA expression than the baT haplotype. This could be due to a slightly higher mRNA stability and half-life, which would result in higher numbers of VDRs being present in the target cell and better response to vitamin D. Although we can assume that mRNA stability differences might be related to allelic differences, the results are not consistent among the studies (30, 41–43).

Low levels of expression, VDR stability and vitamin D3-VDR interaction were related to leprosy and its complications (44). It has been shown that some individuals with normal levels of vitamin D3, but with low levels of VDR protein, had a high bacilloscopic index and type 2 reaction (45). This fact reinforces the results obtained in the present study, which relate both the polymorphism that changes the interaction vitamin D3-VDR (*Fok*I) and the haplotype formed by the *Bsm*I, *Apa*I, and *Taq*I polymorphisms. Although, the *FokI* polymorphism has a higher consistency of results related to VDR and leprosy, the *Bsm*I, *Apa*I, and *Taq*I polymorphisms, especially the haplotype formed by them, should be further investigated in the immunopathogenesis of leprosy.

## Data Availability

This manuscript contains previously unpublished data. The raw data supporting the conclusions of this manuscript will be made available by the authors, without undue reservation, to any qualified researcher.

## Ethics Statement

This work was approved by the Permanent Committee of Ethics in Research with Human Beings of the State University of Maringá (COPEP—UEM) n° 2.424.046/2017.

## Author Contributions

AP performed the experiments and drafted the manuscript. HA, BT, and LM analyzed the data and drafted the manuscript. LV participated in the critical revision of the manuscript. QL and JZ analyzed the data, drafted the manuscript, and participated in the critical revision. AS provided materials and participated in the experimental design. JV designed the study and finalized the manuscript.

### Conflict of Interest Statement

The authors declare that the research was conducted in the absence of any commercial or financial relationships that could be construed as a potential conflict of interest.
